# Supplementary material for: Causal Relationship between Aging and Anorexia Nervosa: A White-Matter-Microstructure-Mediated Mendelian Randomization Analysis
Source: Biomedicines. 2024 Aug 16;12(8):1874. doi: 10.3390/biomedicines12081874 (PMC11351342; doi:10.3390/biomedicines12081874)
Supplement: Supplementary file 1 [file biomedicines-12-01874-s001.zip › Supplementary Figure/Figure S4/Singe SNP plot of MR analysis for TL to ubm-b-2000.pdf]

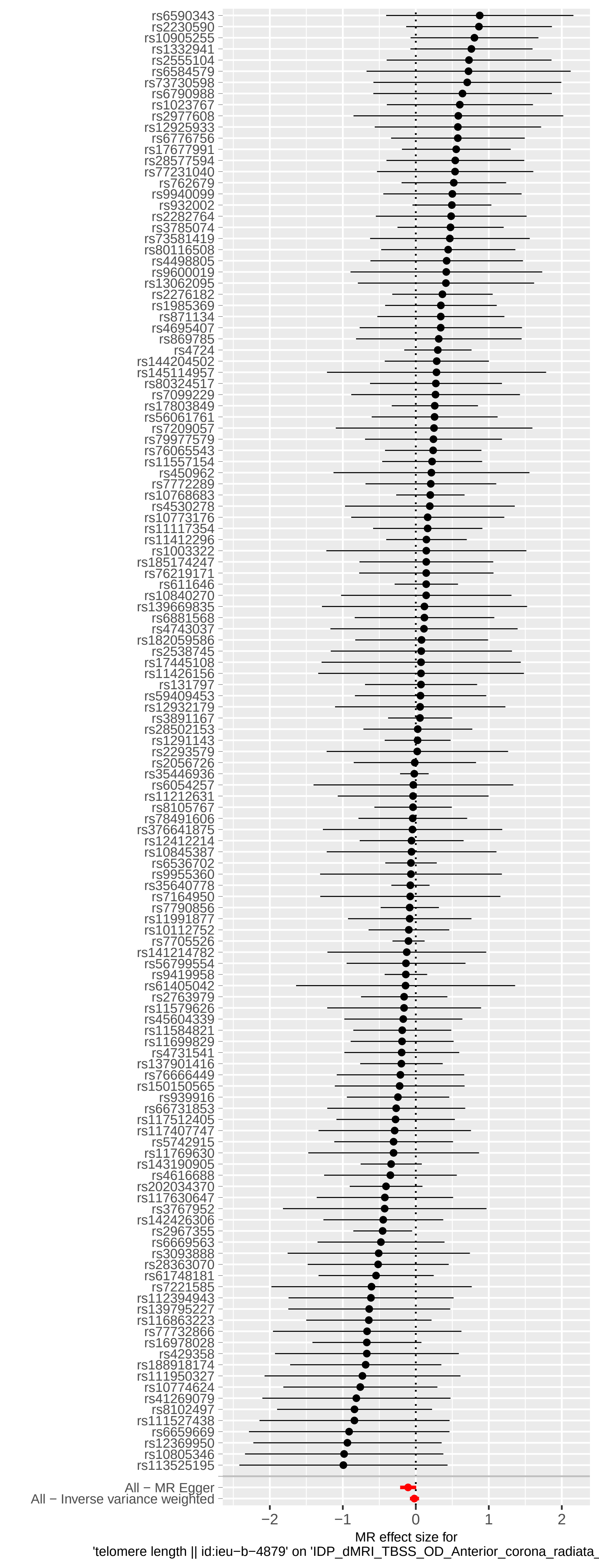
rs6590343
rs2230590
rs10905255
rs1332941
rs2555104
rs6584579
rs73730598
rs6790988
rs1023767
rs2977608
rs12925933
rs6776756
rs17677991
rs28577594
rs77231040
rs762679
rs9940099
rs932002
rs2282764
rs3785074
rs73581419
rs80116508
rs4498805
rs9600019
rs13062095
rs2276182
rs1985369
rs871134
rs4695407
rs869785
rs4724
rs144204502
rs145114957
rs80324517
rs7099229
rs17803849
rs56061761
rs7209057
rs79977579
rs76065543
rs11557154
rs450962
rs7772289
rs10768683
rs4530278
rs10773176
rs11117354
rs11412296
rs1003322
rs185174247
rs76219171
rs611646
rs10840270
rs139669835
rs6881568
rs4743037
rs182059586
rs2538745
rs17445108
rs11426156
rs131797
rs59409453
rs12932179
rs3891167
rs28502153
rs1291143
rs2293579
rs2056726
rs35446936
rs6054257
rs11212631
rs8105767
rs78491606
rs376641875
rs12412214
rs10845387
rs6536702
rs9955360
rs35640778
rs7164950
rs7790856
rs11991877
rs10112752
rs7705526
rs141214782
rs56799554
rs9419958
rs61405042
rs2763979
rs11579626
rs45604339
rs11584821
rs11699829
rs4731541
rs137901416
rs76666449
rs150150565
rs939916
rs66731853
rs117512405
rs117407747
rs5742915
rs11769630
rs143190905
rs4616688
rs202034370
rs117630647
rs3767952
rs142426306
rs2967355
rs6669563
rs3093888
rs28363070
rs61748181
rs7221585
rs112394943
rs139795227
rs116863223
rs77732866
rs16978028
rs429358
rs188918174
rs111950327
rs10774624
rs41269079
rs8102497
rs111527438
rs6659669
rs12369950
rs10805346
rs113525195
All − MR Egger
All − Inverse variance weighted
−2
−1
0
1
2
MR effect size for
'telomere length || id:ieu−b−4879' on 'IDP_dMRI_TBSS_OD_Anterior_corona_radiata_
